# Growth Factor Midkine Aggravates Pulmonary Arterial Hypertension via Surface Nucleolin

**DOI:** 10.1038/s41598-020-67217-w

**Published:** 2020-06-25

**Authors:** Daisuke Kinoshita, Tetsuro Shishido, Tetsuya Takahashi, Miyuki Yokoyama, Takayuki Sugai, Ken Watanabe, Harutoshi Tamura, Satoshi Nishiyama, Hiroki Takahashi, Takanori Arimoto, Takuya Miyamoto, Tetsu Watanabe, Satoshi Kishida, Kenji Kadomatsu, Jun-ichi Abe, Yasuchika Takeishi, Tsuneo Konta, Isao Kubota, Masafumi Watanabe

**Affiliations:** 1 The Department of Cardiology, Pulmonology, and Nephrology, Yamagata University School of Medicine, Yamagata, Japan; 2 Department of Biochemistry, Nagoya University Graduate School of Medicine, Aichi, Japan; 3 Department of Cardiology - Research, Division of Internal Medicine, The University of Texas MD Anderson Cancer Center, Houston, TX US; 4 Department of Cardiology and Hematology, Fukushima Medical University, Fukushima, Japan

**Keywords:** Cardiology, Cardiovascular biology, Cardiovascular diseases, Hypertension

## Abstract

Pulmonary arterial hypertension (PAH) is a progressive fatal disease caused by pulmonary arterial remodeling. Midkine regulates cell proliferation and migration, and it is induced by hypoxia, but its roles in pulmonary arterial remodeling remain unclear. Serum midkine levels were significantly increased in PAH patients compared with control patients. Midkine expression was increased in lungs and sera of hypoxia-induced PAH mice. Hypoxia-induced pulmonary arterial remodeling and right ventricular hypertrophy were attenuated in midkine-knockout mice. Midkine-induced proliferation and migration of pulmonary arterial smooth muscle cells (PASMC) and epidermal growth factor receptor (EGFR) signaling were significantly increased under hypoxia, which also induced cell-surface translocation of nucleolin. Nucleolin siRNA treatment suppressed midkine-induced EGFR activation *in vitro*, and nucleolin inhibitor AS1411 suppressed proliferation and migration of PASMC induced by midkine. Furthermore, AS1411 significantly prevented the development of PAH in Sugen hypoxia rat model. Midkine plays a crucial role in PAH development through interaction with surface nucleolin. These data define a role for midkine in PAH development and suggest midkine-nucleolin-EGFR axis as a novel therapeutic target for PAH.

## Introduction

Pulmonary arterial hypertension (PAH) is characterized by progressive pulmonary arterial remodeling that leads to an increase in pulmonary vascular resistance (PVR)^1,2^. In spite of the advanced medical treatments, the normalization of PVR remains challenging. PAH morbidity and mortality remain high^3^. Excessive proliferation and migration of pulmonary arterial smooth muscle cells (PASMCs) play the central roles in pulmonary arterial remodeling, which is characterized by muscularization of the small vessels that were originally non-muscularized^4,5^. PASMCs and pulmonary arterial endothelial cells obtained from patients with PAH shows marked proliferation in response to growth factors^6,7^. Recently, platelet-derived growth factor (PDGF) and epidermal growth factor (EGF) were reported to be associated with the development of PAH^8,9^. IMPRES trial revealed that the PDGF inhibitor imatinib reduces PVR and increases exercise capacity in patients with PAH^10,11^, but severe adverse effect issues associated with the use of these drugs remain to be resolved^10^.

Midkine is a 13-kDa heparin-binding growth factor involved in various biological processes, including angiogenesis and migration of inflammatory cells^12^. Midkine is expressed in many tissues during the embryonic period, however, its expression in adult tissues, with the exception of kidney, is low^11^. Hypoxia inducible factor 1α (HIF1α) is a direct regulator of midkine, and it exerts its effects by binding to the hypoxia response element at midkine promoter. Normoxic activation of HIF1α causes hyper-proliferation of PASMC-like cancer cells^6,13^. Endothelial dysfunction and ischemia induce the upregulation of midkine expression in the renal tubular cells of chronic kidney disease mouse model and its release to systemic circulation, which results in the activation of angiotensin converting enzyme (ACE) in lungs^14^ and epidermal growth factor receptor (EGFR) in the hearts^15^. Increased serum midkine levels were shown to be independently associated with unfavorable outcomes in patients with heart failure^16^. Moreover, cardiac dysfunction in pressure overload mouse models was reported to induce midkine expression in the lung^17^, and the expression of this gene in respiratory epithelium was demonstrated to be involved in the pathogenesis of pulmonary arterial remodeling^13^. We previously reported that midkine promotes EGFR activation^15^. However, a precise mechanism underlying this process remains unclear.

Nucleolin, reported to be a candidate receptor of midkine^18^, is abundantly expressed in nucleus, mediating ribosome biogenesis and RNA metabolism^19^. Recent studies have demonstrated that it is expressed as well at cell surface and mediates tumorigenesis and cancer-related neoangiogenesis^20,21^. Furthermore, nucleolin was recognized as a EGFR binding partner^22^, stabilizing it and preventing degradation^23,24^. Although the level of surface nucleolin expression in PASMCs remains to be determined, hypoxic exposure induces PASMC phenotypic switch, inducing the proliferation of these cells^25^. Therefore, we hypothesized that hypoxia induces the upregulation of surface nucleolin, which facilitates midkine binding to surface nucleolin and EGFR activation, leading to the development of PAH.

## Results

### Serum midkine levels are increased in PAH patients and mice with hypoxia-induced PAH

**Figure 1:**
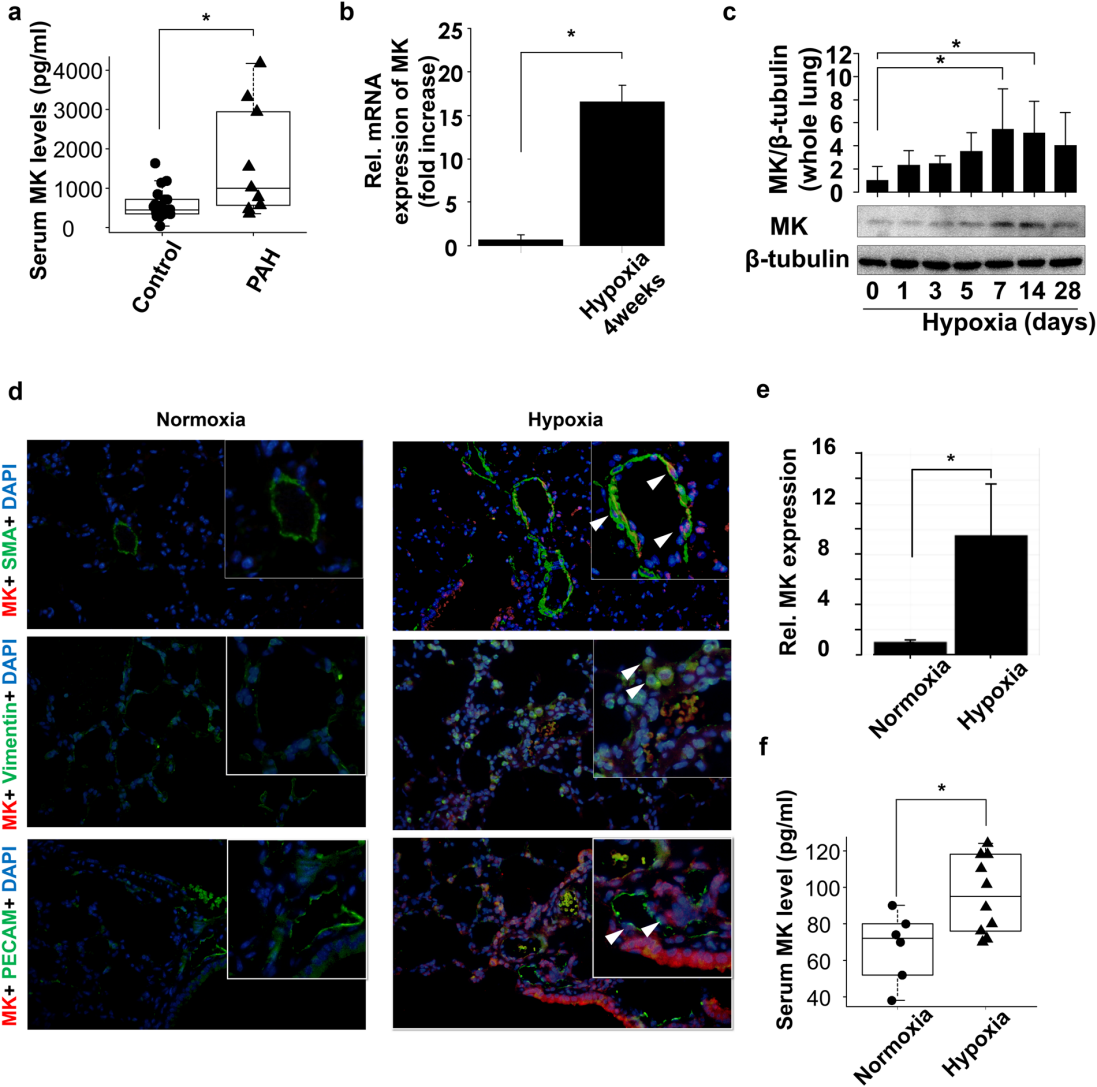
Midkine (MK) expression levels in patients with pulmonary arterial hypertension (PAH) and hypoxia-induced PAH mice. (**a**) Serum MK levels in patients who underwent right-heart catheterization to determine pulmonary vascular resistance (PVR). (**b**) Relative MK mRNA expression in mouse lungs 4 weeks after the exposure to normoxia or hypoxia. (**c**) MK protein expression in mouse lungs during the exposure to hypoxic conditions. Expression levels were normalized to those of β-tubulin. Full-length blots are presented in Supplementary Figure 1 (**d**) Mouse lungs 4 weeks after the exposure to normoxia or hypoxia were stained with antibodies against MK (red), nuclear marker (DAPI, blue), and cell type markers (green) for vascular smooth muscle cell (α-smooth muscle actin (SMA)), stromal cell (vimentin), and endothelial cell (platelet endothelial cell adhesion molecule (PECAM)). Arrowheads in each image show both MK and the cell type marker positive cells. (**e**) Fluorescent intensity was quantified and normalized to the background intensity by using ImageJ software (version 1.42; https://imagej.nih.gov/ij/). (**f**) Serum MK concentrations in mice exposed to normoxia or hypoxia were evaluated. Data are expressed as mean ± SE (n = 6–10 mice per group). *ANOVA post-hoc Tukey’s honest significant difference, P < 0.05 *vs*. the indicated control.

We measured serum midkine levels in patients with PAH or those with chronic heart failure. Baseline patient characteristics are presented in Table 1. Serum midkine levels were significantly increased in patients with PAH in comparison with those with heart failure without increased PVR (Fig. 1a). Next, we investigated midkine expression levels in hypoxia-induced PAH mice model. Midkine mRNA levels were shown to be significantly upregulated in the lungs of these mice (Fig. 1b). Midkine expression levels in the lung during hypoxia were shown to increase in a time-dependent manner (Fig. 1c). Midkine expression was observed in respiratory epithelium, arteriole, and vascular adventitia in hypoxia-induced PAH mice lung (Fig. 1d,e). Serum midkine levels were increased in PAH mice compared with those in the sham control as well (Fig. 1f).

**Table 1:** Baseline characteristics among groups.

	All Patients (n = 31)	Control (n = 22)	PAH (n = 9)	P value
Age, years	65 ± 2	66 ± 2	66 ± 3	0.328
Gender (male), n (%)	327 (87)	19 (86)	8(89)	0.12
NYHA I/II/III/IV, n	15/7/7/2	15/3/4/0	0/4/3/2	0.18
Clinical classification, Group	1/2/3/4/5		1/0/1/1/6	
Serum Creatine, mg/dL	0.88 ± 0.05	0.86 ± 0.05	0.93 ± 0.1	0.542
Mean PAP, mmHg	19.77 ± 1.73	15.09 ± 1.01	31.22 ± 2.99	<0.0001
PCWP, mmHg	8.5 ± 0.92	7.95 ± 0.92	9.9 ± 2.3	0.351
PVR, wood unit	2.36 ± 0.28	1.8 ± 0.16	4.4 ± 0.72	0.07
AF, n (%)	12 (39)	9 (41)	5 (56)	0.232
DM, n (%)	9 (29)	5 (23)	4 (44)	0.241
HT, n (%)	15 (48)	41.86 ± 1.67	6 (67)	0.12
LAD, mm	42.93 ± 1.43	41.86 ± 1.67	45.44 ± 2.72	0.209
LVEF, %	53.93 ± 3.38	57.33 ± 3.9	46 ± 6.17	0.292
Hemoglobin, g/dL	14.22 ± 0.34	14.37 ± 0.27	13.83 ± 0.99	0.48
BNP, pg/ml	443.52 ± 99.59	367.53 ± 126.62	629.29 ± 138.39	0.239
MK, ng/ml	884.91 ± 169.77	565 ± 77.43	1666.94 ± 475.49*	0.002

Data are presented as means ± SD or %; NYHA, New York heart association functional class; PAP, pulmonary arterial pressure; PVR, pulmonary vascular resistance; AF, atrial fibrillation; PCWP; pulmonary capillary wedge pressure; DM, diabetes mellitus; HT, hypertension; HL, hyperlipidemia; LAD, left atrial diameter; LVEF, left ventricular ejection fraction; BNP, brain natriuretic peptide; MK, midkine. P value was calculated by ANOVA, * vs Control.

### Midkine deficiency ameliorates rate pulmonary arterial remodeling in PAH mice

**Figure 2:**
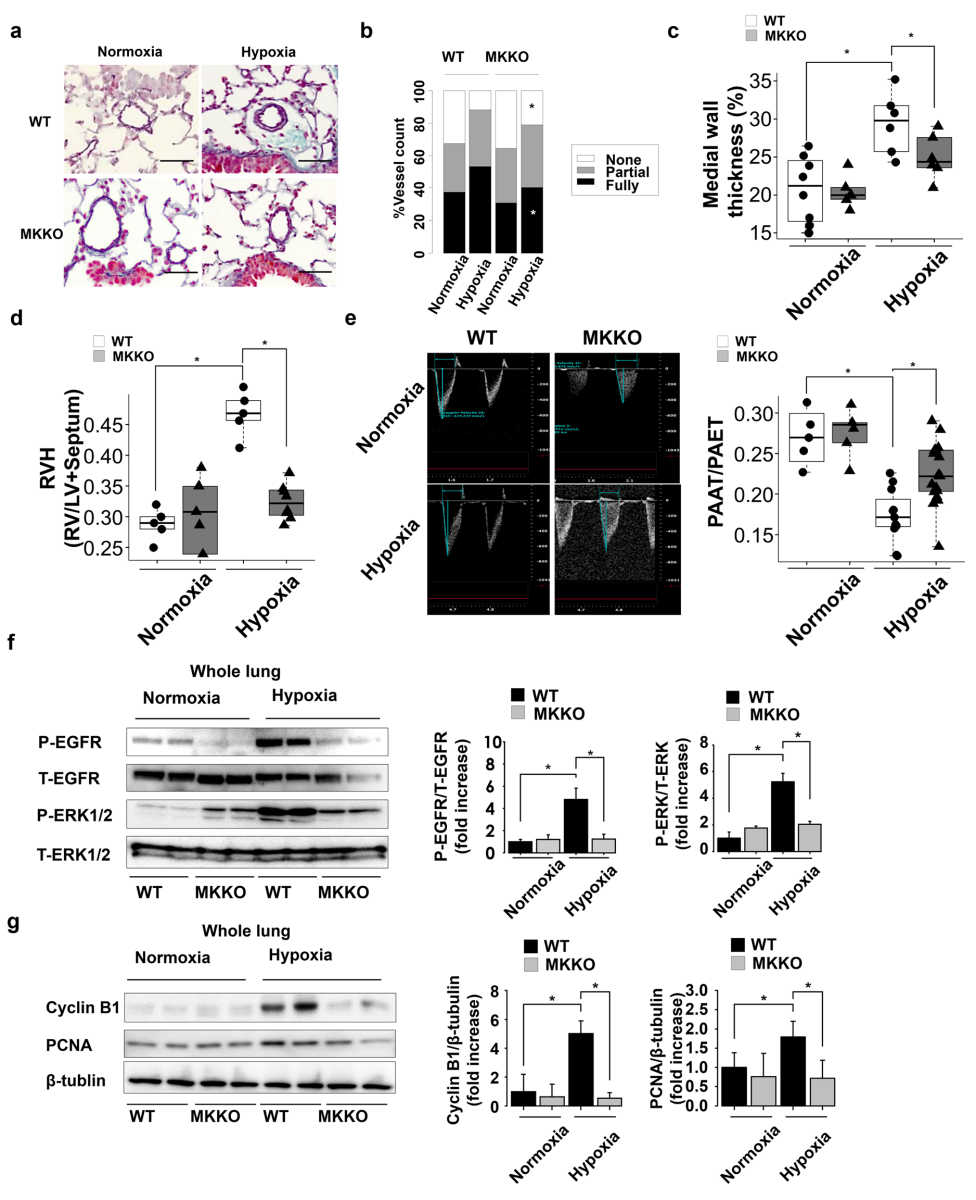
Systemic midkine (MK) knockdown effects on pulmonary arterial remodeling in chronic hypoxia-induced pulmonary arterial hypertension (PAH) mice. (**a**) Representative Elastica-Masson staining images of mouse lung transverse sections at 4 weeks after the exposure to normoxia or hypoxia. Scale bars, 50 µm. (**b**) Degree of muscularization of pulmonary arterial vessels (outer diameter 20–100) in mice exposed to normoxia or hypoxia (n = 5–9 each). WT, *wild-type* mice; MK-KO, midkine knockdown mice. (**c**) Pulmonary arterial remodeling evaluated by the percentage of medial wall thickness (n = 5–9 each). (**d**) Right hypertrophy [ratio of right ventricle (RV)/left ventricle plus septum (LV + S)] (n = 5–9 each). (**e**) Pulmonary artery acceleration time (PAAT)/ pulmonary artery ejection time (PAET), determined by echocardiography (n = 5–15 each). (**f**) Western blots analysis of EGFR and ERK1/2 phosphorylation rates in mouse lungs (n = 5). Full-length blots are presented in Supplementary Figure 2. (**g**) Western blots analysis for Ccnb1, PCNA, and β-tubulin levels in mouse lungs (n = 5). Full-length blots are presented in Supplementary Figure 3. *ANOVA post-hoc Tukey’s honest significant difference, P < 0.05, compared with the indicated control.

We exposed WT and MK-KO mice to chronic hypoxia for 4 weeks to investigate the impact of midkine on the pulmonary arterial remodeling. There were no significant differences in the pulmonary arterial findings between WT and MK-KO mice under normoxic conditions. The ratio of fully-muscularized vessels and medial wall thickness were shown to increase following the exposure to hypoxia in the WT mice. Midkine deficiency was shown to significantly suppress pulmonary arterial remodeling rate (Fig. 2a–c). The increased ratio of RV/LV + S and the decreased ratio of pulmonary artery acceleration time to pulmonary artery ejection time, determined by echocardiography, were also decreased in MK-KO mice, in comparison with those in the WT mice (Figs. 2d,e). Hypoxia-induced phosphorylation of EGFR and extracellular signal-regulated kinase (ERK) 1/2 was shown to be suppressed in MK-KO mice, compared with that in the WT mice (Fig. 2f). The expression of cell proliferation markers, Cyclin B1 and proliferation cell nuclear antigen (PCNA), was significantly increased in the hypoxic WT mice, but considerably attenuated in the MK-KO mice (Fig. 2g).

### Hypoxic conditions enhance midkine-induced PASMCs migration and proliferation

**Figure 3:**
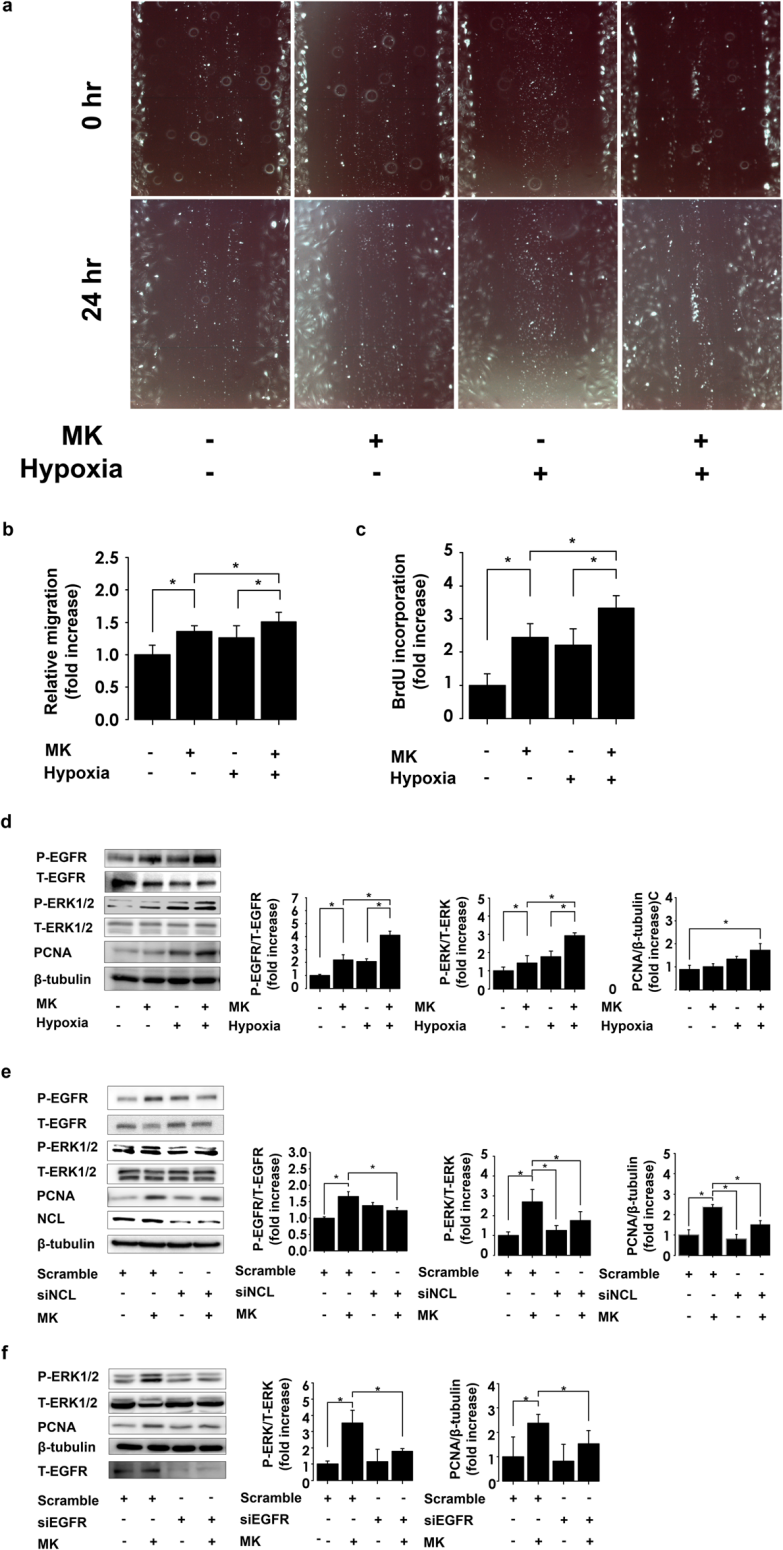
Midkine (MK) effects on pulmonary arterial smooth muscle cell (PASMC) migration and proliferation mediated through nucleolin/EGFR pathway. (**a**) Representative scratch assay images of PASMCs treated with vehicle or MK under normoxic or hypoxic conditions. (**b**) Quantification of the scratch area coverage by the migrating cells using ImageJ software (version 1.42; https://imagej.nih.gov/ij/). Migration levels were normalized to that of the control (n = 18). (**c**) PASMC proliferation assay, after the stimulation with or without MK (n = 18). (**d**) Representative ERK1/2, EGFR and proliferating cell nuclear antigen (PCNA) expression results in PASMCs exposed to hypoxia or normoxia and/or MK treatment. Full-length blots are presented in Supplementary Figure 4. (**e**) Representative images of EGFR, ERK1/2, PCNA, nucleolin (NCL), and β-tubulin expression in PASMCs, and their quantification, following the treatment of PASMCs with MK in cells where nucleolin expression was inhibited (n = 6). Full-length blots are presented in Supplementary Figure 5. (**f**) Representative image of western blots of ERK1/2, PCNA, β-tubulin, and EGFR. PASMC was treated with midkine under downregulation of EGFR using siRNA. Silencing of EGFR suppressed the midkine-induced ERK1/2 phosphorylation and PCNA expression. n = 6. Full-length blots are presented in Supplementary Figure 6. * ANOVA post-hoc Tukey’s honest significant difference, P < 0.05 compared to indicated control.

Next, we performed scratch assay to evaluate the effects of midkine on the migration of PASMCs, and showed that the treatment with recombinant midkine significantly induces the migration of these cells, compared with that of the vehicle control cells. Interestingly, midkine-induced PASMC migration was significantly enhanced by hypoxic conditions, compared with the effects of normoxia (Fig. 3a,b). The incorporation of 5-bromo-2-deoxyuridine (BrdU) revealed that midkine significantly induces PASMC proliferation, compared with that of the control cells, which was further enhanced by hypoxic conditions (Fig. 3c).

### Nucleolin and EGFR mediate midkine-induced alterations

EGFR and ERK1/2 were shown to be phosphorylated at 3 min and 10 min, respectively, following the treatment of PASMCs with midkine. This EGFR signaling activation was significantly promoted by the exposure to hypoxia (Fig. 3d). We used nucleolin-specific small interfering RNA (siRNA) to silence nucleolin expression, and stimulated PASMCs using the recombinant midkine, which demonstrated that the silencing of this gene leads to a significant suppression of midkine-induced EGFR and ERK1/2 phosphorylation (Fig. 3e). Midkine induced-PCNA expressions were similarly increased by the exposure to hypoxia, and suppressed by nucleolin depletion (Fig. 3d,e). Additionally, we showed that EGFR-specific siRNA treatment significantly suppressed midkine-induced ERK1/2 phosphorylation and PCNA expression (Fig. 3f).

### Hypoxic conditions induce nucleolin translocation to the cell surface

**Figure 4:**
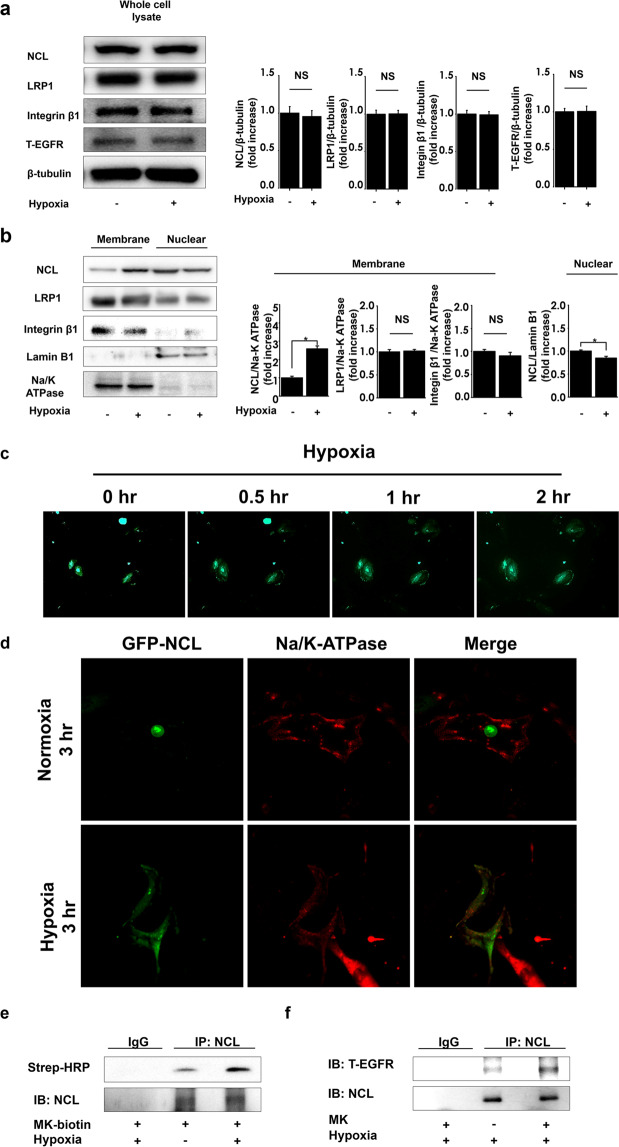
Hypoxia induces cell surface translocation of nucleolin under hypoxic conditions. (**a**) PASMCs were lysed following the exposure to normoxia or hypoxia (FiO_2_ 0.03) for 1 h. Nucleolin (NCL), low density lipoprotein related protein 1 (LRP1), integrin β1, epidermal growth factor receptor (EGFR), and β-tubulin expression levels were evaluated. Representative images of western blot, and their quantification (n = 4). Full-length blots are presented in Supplementary Figure 7. (**b**) Nucleolin (NCL), LRP1, integrin β1, lamin B, and Na/K-ATPase expression in nuclear or membrane fractions of pulmonary arterial smooth muscle cells (PASMCs) after their exposure to hypoxia or normoxia, and their quantification (n = 6). Full-length blots are presented in Supplementary Figure 8. (**c**) The distribution of green fluorescent protein (GFP)-tagged nucleolin was observed under hypoxia, using time-lapse microscopy. (**d**) Confocal laser microscopy image of GFP-tagged nucleolin and Na/K-ATPase, under hypoxic conditions. (**e**) PASMCs were treated with biotinylated midkine (MK) or vehicle, and exposed to normoxia or hypoxia, and midkine-nucleolin binding was analyzed. Full-length blots are presented in Supplementary Figure 9. (**f**) NCL-EGFR interactions in PASMCs treated with MK or vehicle, and exposed to hypoxia. Strep-HRP, horseradish peroxidase conjugate streptavidin. Full-length blots are presented in Supplementary Figure 10.

We observed that the exposure to hypoxic conditions did not alter the expression of nucleolin, EGFR, and other midkine receptor candidates, including low density lipoprotein related protein 1 (LRP1) and integrin β1 in PASMCs (Fig. 4a). However, the expression of nucleolin in the membrane fraction of these cells was significantly increased after hypoxia exposure, while nuclear nucleolin levels were significantly decreased. The expression of LRP1 and integrin β1 in both membrane and nuclear fractions did not change (Fig. 4b). Next, we observed the localization of green fluorescent protein (GFP)-tagged nucleolin during cell exposure to hypoxia using time-lapse imaging. GFP-tagged nucleolin was observed to be predominantly expressed in the cell nuclei under the normoxic conditions. However, following the exposure of cells to hypoxic conditions, GFP-nucleolin expression decreased in nuclei and increased in the extranuclear lesions, in a time-dependent manner (Fig. 4c). Confocal laser microscopy revealed that GFP-tagged nucleolin co-localized with cell surface protein Na/K-ATPase during hypoxia (Fig. 4d). Next, we examined the impact of hypoxic exposure on midkine-nucleolin interactions. Immunoprecipitation assay revealed that midkine interacts with nucleolin, and this interaction was enhanced by hypoxic exposure (Fig. 4e). Further, we observed that nucleolin interacts with EGFR as well, and although this interaction was shown to be weak even under hypoxic conditions, midkine treatment led to its increase (Fig. 4f).

### AS1411 inhibits midkine-induced cellular processes

**Figure 5:**
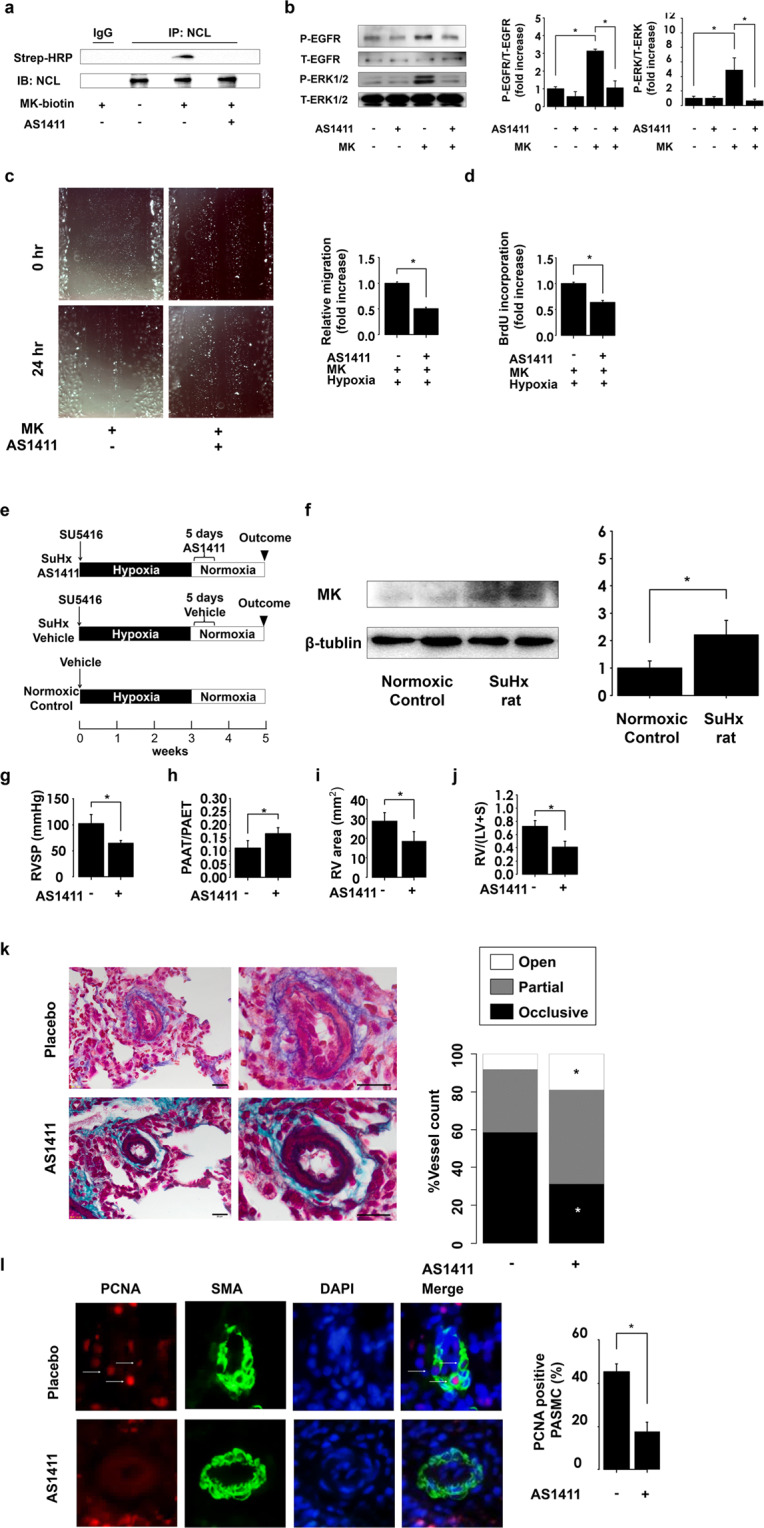
AS1411 suppresses the development of pulmonary arterial hypertension (PAH) by inhibiting midkine (MK)-nucleolin (NCL)-EGFR axis. (**a**) Pulmonary arterial smooth muscle cells (PASMCs) were treated with vehicle or AS1411, which was followed with the treatment with biotinylated MK. Full-length blots are presented in Supplementary Figure 11. (**b**) Phosphorylated ERK1/2 and EGFR (p-ERK1/2 and p-EGFR) and total ERK1/2 and EGFR (t-ERK1/2 and t-EGFR) expression levels in PASMCs treated with MK after AS1411 pretreatment. Full-length blots are presented in Supplementary Figure 12. (**c**) Representative images of MK-induced migration of PASMCs after the pretreatment of cells with placebo and AS1411, and its quantification using ImageJ software (version 1.42; https://imagej.nih.gov/ij/). (**d**) MK-induced proliferation of PASMCs under hypoxic conditions. **(e**) Study design. Rats were divided into three groups, and the rats were treated with AS1411 (10 mg/kg/day) or the vehicle for consecutive 5 days after the establishment of PAH. Normoxic control was treated with vehicle and exposed to hypoxic conditions for 3 weeks, which was followed by the re-exposure to normoxia. (**f**) Western blot analysis midkine (MK) and β-tubulin expression in lung homogenates, and their quantification. Full-length blots are presented in Supplementary Figure 13. (**g**) Right ventricular systolic pressure (RVSP) was assessed following the treatment with AS1411 (n = 4). (**h**) The ratio of pulmonary artery acceleration time (PAAT) and pulmonary artery ejection time (PAET), assessed by echocardiography (n = 4). (**i**) Analysis of RV chamber area was assessed at the papillary muscle level using B-mode (n = 4). (**j**) RV hypertrophy assessed by RV/left ventricle plus septum (LV + S). (**k**) Representative images of Elastica-Masson staining demonstrating the occlusive pulmonary arterial remodeling in Sugen/hypoxia (SuHx) rats. Scale bar, 20 µm; (n = 5). (**l**) PCNA (red), α-smooth muscle actin (SMA, green), and DAPI (Blue) lung section immunostaining in SuHx. PCNA-positive smooth muscle cells were counted in at least 20 distal pulmonary arteries (external diameter <50 µm); (n = 5). *ANOVA post-hoc Tukey’s honest significant difference, P < 0.05, compared with the indicated control.

Immunoprecipitation assay results demonstrated that the nucleolin inhibitor AS1411 inhibits the interactions between nucleolin and biotinylated recombinant midkine (Fig. 5a). AS1411 significantly inhibited midkine-induced EGFR and ERK1/2 phosphorylation, compared with that in the control cells (Fig. 5b). Midkine-induced migration and proliferation of PAMSCs under the hypoxic conditions were significantly suppressed following the pretreatment with AS1411 (Fig. 5c,d).

### AS1411 ameliorates the development of Sugen/hypoxia (SuHx)-induced PAH

We investigated whether AS1411 can suppress the pulmonary arterial remodeling and development of PAH in SuHx-triggered PAH rat model, because this model is characterized by severe PAH, and has properties similar to the pathological features of human PAH, which is irreversible and progressive under normoxic conditions^26^. Rats were divided into three groups, as shown in Fig. 5e. SuHx rats were treated with AS1411 or vehicle after the establishment of PAH as previously described^27^. Western blot analysis demonstrated that midkine expression increases in the lungs of SuHx rats, compared with that in the normoxic control (Fig. 5f). AS1411 treatment led to a significant decrease in the RV systolic pressure in SuHx rats, compared with that in the controls (Fig. 5g). The decreased ratio of pulmonary artery acceleration time/pulmonary artery ejection time determined was shown to be attenuated as well after the treatment with AS1411, compared with that of the placebo control (Fig. 5h). Enlarged RV chamber area and RV hypertrophy were significantly suppressed in AS1411-treated rats (Fig. 5i,j). Histological analysis revealed that AS1411 led to a significant decrease in the occlusive vessel ratio and PCNA expression (Fig. 5k,l).

## Discussion

Here, we demonstrated that midkine was shown to induce the proliferation and migration of PASMCs and midkine deficiency attenuated pulmonary arterial remodeling in hypoxia-induced PAH mice. Hypoxia induced the translocation of nucleolin to the cell surface, and enhanced midkine-induced activation of EGFR signaling. Silencing of nucleolin expression by siRNAs and AS1411 suppressed midkine-induced EGFR signaling and PASMC proliferation, and this inhibitor was shown to attenuate the development of PAH by suppressing pulmonary arterial remodeling.

The pathogenesis of PAH involves a combination of factors, including gene mutation, inflammation, pulmonary endothelial dysfunction, and pulmonary vasculature cell proliferation. PASMCs obtained from patients with PAH are more sensitive to growth factors than those from the control subjects, and sustained growth signaling causes the excessive proliferation of PASMCs^7^. EGFR signaling is a major contributor of PASMC proliferation, migration, and survival, and an EGFR inhibitor was shown to ameliorate pulmonary arterial remodeling in monocrotaline-induced PAH rat^28^. Although circulating midkine was reported to exacerbate cardiac remodeling via EGFR signaling^15^, the relationship between serum midkine levels and pulmonary arterial remodeling has not been elucidated. Here, we demonstrated that serum midkine levels in patients with PAH are considerably higher than in the patients with heart failure without pulmonary arterial remodeling. The upregulation of serum midkine levels and EGFR signaling activation were observed in hypoxia-induced PAH mice, and midkine deficiency attenuated EGFR activation and pulmonary arterial remodeling. These results suggest a crucial role of midkine in the development of PAH.

Many studies demonstrated that midkine plays important roles in cell migration and proliferation^29–31^. However, the mechanism underlying these processes has not been clarified. Although midkine upregulation has been demonstrated as a predictive marker for poor outcomes in patients with cancer and chronic heart failure, the cell proliferative effects of midkine does not always promote pulmonary arterial remodeling in these patients^16,32,33^. Therefore, we hypothesized that the increased expression or distribution change of midkine receptors was involved in the development of PAH. We examined the expression levels and distribution of candidates receptors for midkine, such as LRP1, integrin β1, and surface nucleolin^15^. Casttelano *et al*. reported that exposure to hypoxia induces the expression of LRP1 in human coronary arterial smooth muscle cells (hCASMC)^34^. However, we did not observe similar changes in this study, which may be explained by the different cell types and hypoxic conditions we used, since the effects of hypoxia on PASMCs were reported to differ from those observed using hCASMC^35^. However, we observed that hypoxia leads to the alterations in the subcellular distribution of nucleolin, which facilitates midkine-induced activation of EGFR signaling. To the best of our knowledge, this study is a first report demonstrating the role of surface nucleolin in midkine-induced EGFR activation and subsequent PASMC proliferation and PAH development. Several mechanisms of abnormal EGFR activation in different cells have been observed, such as due to autocrine/paracrine ligand loops, gene mutation, overexpression and impaired degradation of EGFR, and the heterodimerization of EGFR with ErbB2^36^. Similar to that observed in a previous report^37^, there was no change in EGFR expression in PASMCs grown under hypoxic conditions. Moreover, as observed in the previous studies, using cancer cell lines, we observed a weak interaction between nucleolin and EGFR^23^, although nucleolin was expressed on the surface of PASMCs under hypoxic conditions. This aberrant interaction required midkine stimulation. Furthermore, the silencing of nucleolin and the inhibition of midkine binding to nucleolin attenuated midkine-induced EGFR activation, indicating that midkine may facilitate EGFR activation via nucleolin. Guanine adenine-rich domain at C terminal portion of nucleolin was demonstrated to be a lesion responsible for the interaction with both midkine and EGFR^18,22^. We observed that EGFR activation had a trend toward an increase by silencing nucleolin expression, although the role of nucleolin in EGFR activation remains to be elucidated. Wolfson *et al*. demonstrates that overexpressed nucleolin promotes ligand-independent EGFR activation^38^. In contrast, Reyes-Reyes el al reported that nucleolin depletion also activates EGFR via Rac1 activation^39^. It is possible that the difference in expression levels of surface nucleolin cause this discrepancy. Although our data revealed the involvement of nucleolin in midkine-induced EGFR activation, further study is needed to investigate the role of midkine in the interaction between nucleolin and EGFR activation.

**Figure 6:**
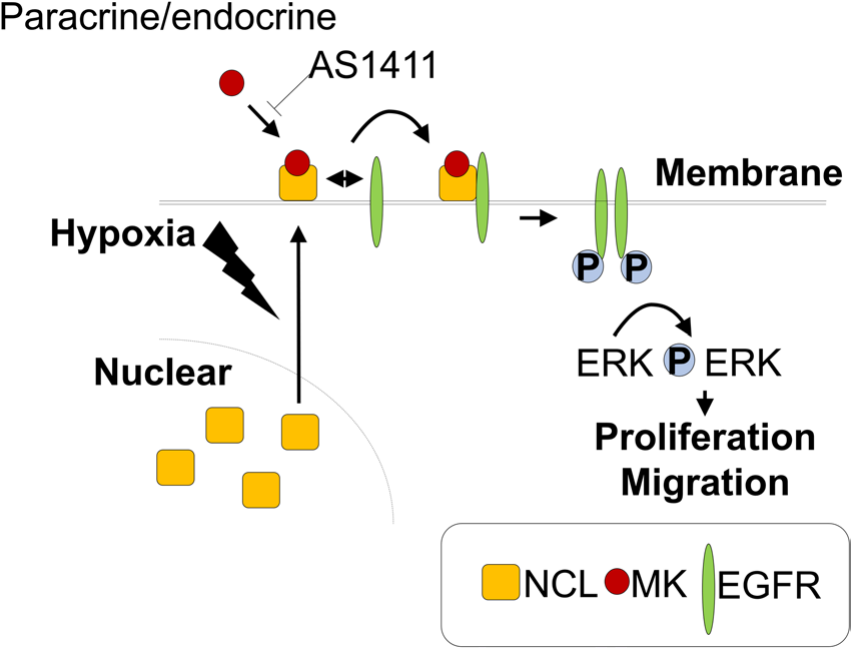
Proposed mechanism underlying midkine (MK) effects on proliferation and migration of pulmonary arterial smooth muscle cells (PASMCs). MK contributes to the development of pulmonary arterial remodeling via cell surface nucleolin translocation under hypoxic conditions, and promotes the activation of epidermal growth factor receptor (EGFR) by inducing the interactions between nucleolin and EGFR. The inhibition of the MK binding to nucleolin by AS1411 suppresses the proliferation of PASMCs and attenuates the development of pulmonary arterial hypertension **(**PAH).

The schematic illustration of the pathway suggested by the results of this study is presented in Fig. 6. Secreted midkine binds to nucleolin on the cell surface, which results in the activation of EGFR and the downstream ERK1/2. Hypoxia promotes cell surface translocation of nucleolin, facilitating midkine binding to the surface nucleolin, inducing EGFR signaling activation. AS1411 is an aptamer that specifically binds to the surface and cytoplasmic nucleolin molecules, and a promising agent with demonstrated clinical efficacy and low toxicity, and due to these facts, AS1411 was studied in several human clinical anti-cancer trials^40,41^. Since this inhibitor does not affect nucleolin expression and stability^40^, the precise mechanism underlying the observed effects has not been elucidated to date. However, we found that AS1411 strongly inhibits midkine binding to nucleolin, which consequently affects cellular processes.

In this study, we demonstrated that midkine contributes to the development of pulmonary arterial remodeling. Additionally, we elucidated the mechanism underlying midkine-induced proliferation and migration of PASMC via surface nucleolin, followed by EGFR activation. The results of our study demonstrated that midkine plays an important role in the pathogenesis of PAH, and midkine-nucleolin-EGFR axis may represent a novel therapeutic target for PAH.

## Limitations

We did not identify what cells were responsible for midkine secretion. Midkine is possibly secreted from various cells such as endothelial cell and alveolar epithelial cell^30,42^, and affects these cells functions. In the current study, we particularly focused the circulating midkine roles in PAH instead of detecting the responsible cells to secrete midkine. We did not clarify the effects of midkine on cells other than PASMC in the development of PAH. Further study is required to detect the cell or tissue to secrete the midkine in PAH models. The number of human PAH samples was small and severity of PAH was relatively mild compared with other studies. We could not measure the pulmonary arterial pressure of mice because of technical difficulty. However, many studies do not perform catheterization to evaluate the pulmonary arterial pressure in PH mice model^43,44^, because pulmonary arterial pressure can be estimated by echocardiographic parameter such as pulmonary acceleration time^45^.

## Methods

### Ethics statement

All experimental procedures were performed according to the animal welfare regulations of Yamagata University School of Medicine, and the study protocol was approved by the Animal Subjects Committee of Yamagata University School of Medicine. The investigation was confirmed to Guide for the Care and Use of Laboratory Animals published by the US National Institutes of Health (NIH Publication, 8^th^ Edition, 2011). For the experiments involving human samples, written informed consent was obtained from all the patients before the study. The study was performed in accordance to the Helsinki Declaration, and the protocol was approved by the Ethical Review Committee of Yamagata University School of Medicine.

### Materials and reagents

Recombinant human midkine was purchased from Peptide Institute (catalog No. 4298-V, Osaka, Japan); antibodies against extracellular signal regulate kinase (ERK, catalog No. 9212 s) 1/2, phospho-ERK1/2 (p-ERK1/2, catalog No. 9101 s), phospho-epidermal growth factor receptor (P-EGFR, catalog No. 2234 s), β-tubulin (catalog No. 2146 s), and Na/K-ATPase (catalog No. #3010) were purchased from Cell Signaling Technology (Danvers, MA, USA). Antibodies against midkine (catalog No. sc-20715), EGFR (catalog No. sc-03), integrin β1 (catalog No. sc6622), proliferation cell nuclear antigen (PCNA, catalog No. sc-7907), lamin B1 (catalog No. sc-20682), and Cyclin B1 (catalog No. sc-245) were purchased from Santa Cruz (Dallas, TX, USA). Antibody against platelet endothelial cell adhesion molecule (PECAM, catalog No. 550274) was purchased from BD pharma (Franklin Lakes, NJ, USA). Anti-lipoprotein related protein 1 (LRP1, catalog No. ab92544), vimentin (catalog No. ab92547) and anti-nucleolin (catalog No. ab72558) antibodies were purchased from Abcam (Cambridge, MA, USA). AS1411 (5′-GGTGGTGGTGGTTGTGGTGGTGGTGG-3′, nucleolin aptamer) was purchased from Invitrogen (Carlsbad, CA, USA). Nucleolin (catalog No. 4390771) small interfering RNA (siRNA) was purchased from Abcam. EGFR (catalog No. 6482 s) siRNA was obtained from Cell Signaling Technology, while the negative-control (catalog No. 12935) siRNA was obtained from Invitrogen.

### Animal models

Midkine knockout (MK-KO) mice, with C57BL/6 background, were established as previously reported^15^. Age-matched 10-week-old male *wild-type* (WT) mice and MK-KO mice were exposed to hypoxic or normoxic conditions during 4 weeks. Briefly, hypoxic mice were placed in a chamber with gas-mixture of 10% O_2_ and 90% N_2_ by adsorption-type oxygen concentrator that utilizes the exhaust air (Teijin, Tokyo, Japan). We generated sugen-hypoxia (SuHx) induced PAH rats, which were reported to resemble severe human PAH^46^. Sprague-Dawley rats (200–250 g body weight) received a single subcutaneous injection (s.c) of Sugen 5416 (AdooQ BioScience, CA, USA, 20 mg/kg, catalog No. A12437-50) and were exposed to normobaric hypoxia as previously reported^46^. After 3 weeks of hypoxia, the animals were returned to normoxic conditions and randomly assigned to either the treatment group, which received AS1411 (10 mg/kg) or saline for 5 consecutive days, and housed under normoxic conditions for additional 2 weeks^27^. Five weeks after the treatment with Sugen 5416, animals were anesthetized with intraperitoneal injection of ketamine (80 mg/kg per hour) and xylazine (8 mg/kg per hour) mixture, in order to perform transthoracic echocardiography and right heart catheterization^47^. Right jugular vein was cannulated and optic fiber pressure catheter (420LP, SAMBA Sensors, Sweden) was advanced into right ventricle (RV) to measure right ventricular systolic pressure (RVSP). For the assessment of echocardiography, the ratio of pulmonary artery acceleration time to pulmonary artery ejection time and right ventricular area were assessed using a Vevo2100 (VisualSonics, Toronto, ON, Canada).

Pulmonary arterial remodeling was assessed as described previously^48^. The lung was fixed with 4% paraformaldehyde in phosphate-buffered saline (PBS) for 24 h, embedded in paraffin, and cut serially. Elastica-Masson staining was performed to measure the medial wall thickness of distal pulmonary vessels (20–100 μm) along shortest external diameter by using ImageJ software (National Institutes of Health) under a microscope (BX50; Olympus, Tokyo, Japan). Medial wall thickness was expressed as follows: Percent of wall thickness = [(external diameter–internal diameter)/external diameter] × 100. Vessels were categorized as fully-muscularized (>70% medial coat of muscle), partially-muscularized (5–70% medial coat of muscle), and non-muscularized vessels (<5% medial coat of muscle). In SuHx rat model, a quantitative analysis of luminal obstruction was performed in small pulmonary arteries (outer diameter <50 µm). Vessels were evaluated for occlusive lesions on Elastica-Masson slides and classified as follows: no evidence of neointimal thickness (Open); partial (<50%) luminal occlusion (Partial); and full (>50%)-luminal occlusion (Occlusive)^48^. For each animal, at least 100 vessels were measured at ×400 magnification in a blind manner. For the assessment of right ventricular hypertrophy, the RV was separated from the left ventricle (LV) and septum (S), and RV/(LV + S) ratio was calculated.

### PASMC primary culture

Rat pulmonary arterial smooth muscle cells (PASMCs, catalog No. R352–05a) were obtained from Cell Applications, Inc. (San Diego, CA, USA). The cells were stored in −80 °C and cultured according to the provider’s instructions. PASMCs at passages 5 to 10, and at 70–90% confluence were used for experiments.

### Protein extraction and western blotting

Total proteins were extracted from the mouse lung and PASMCs using ice-cold RIPA buffer^48^. The protein concentration of each sample was determined using the BCA protein assay (BioRad Laboratories, Inc., Hercules, CA, USA). Equal amounts of protein were electrophoresed on 6–14% sodium dodecyl sulfate (SDS)-polyacrylamide gels and electrotransferred onto polyvinylidene difluoride membranes. Membranes were blocked by 2% bovine serum albumin (BSA) with TBS-T (20 mM Tris-HCL, pH 7.4, containing 150 mM NaCl, 0.1% Tween) and then probed with primary antibodies diluted in TBS-T. After incubation with horseradish peroxidase (HRP) -conjugated secondary antibodies diluted in TBS-T containing 5% milk, immunoreactive bands were detected using ECL kit (Amersham Biosciences, Piscataway, NJ, USA).

### Real-time reverse transcription polymerase chain reaction (RT-PCR)

Total RNA was extracted from the animal tissue using TRIzol^49^. First-strand cDNA was synthesized from a 1-µg sample using oligo (dt) primers (Sigma-Aldrich, MO, USA) and Superscript III reverse transcriptase. Real-time polymerase chain reaction (RT-PCR) was performed with Fast SYBR Green master mix in a 20-µL volume reaction using 7500 Fast RT-PCR system (Thermo Fisher Scientific, IL, USA). The following primers were used: for midkine, 5′-accgaggcttcttccttctc-3′ (forward), and 5′-ggctccaaattccttcttcc-3′ (reverse); for β-2 microglobulin (B2M), 5′-agcccaagaccgtctactgg-3′ (forward), and 5′-ttctttctgcgtgcataaattg-3′ (reverse). Gene expression levels were normalized to the level of B2M (Gene bank No: Midkine, NM_001012335.1; B2M, NM_009735).

### Enzyme-linked immunosorbent assay (ELISA)

Serum midkine levels of mice and human specimens were assessed by ELISA (for mice (catalog No. SEA631Mu), Cloud-Clone Corp. Houston, TX, USA; for humans (catalog No. OK-6149), Assay Biotech, Sunnyvale, CA USA,), according to the manufacturer’s^16^ instructions. Blood samples were obtained from chronic hypoxia-induced pulmonary arterial hypertension (PAH) mice and consecutive 43 human patients with suspected heart failure due to non-ischemic cardiomyopathy who underwent right catheterization in our hospital from January 2008 to December 2009. We excluded the 12 patients with increased pulmonary precapillary wedge pressure (clinical classification group 2), and patients were divided into two groups according to presence of pulmonary vascular remodeling defined as increased PVR (≧3 wood unit).

### Immunofluorescence

Lung sections were treated with a blocking agent before the incubation with a primary antibody against midkine, and the sections were incubated with primary antibody overnight. This was followed by the treatment with Alexa-555 anti-rabbit secondary antibody (Invitrogen, catalog No. 43957 A) and fluorescein isothiocyanate (FITC) conjugated anti-smooth muscle actin antibody (catalog No. F3777) for 1 h at room temperature, which was accompanied by the incubation with 4′, 6-diamidino-2-phenylindole (DAPI (catalog No. 135–1303); Lonza, Bazel Switzerland) before mounting. The slides were observed under an immunofluorescence microscope (DP-70, Olympus)^15^. Fluorescent intensity was quantified and normalized to the background intensity using ImageJ software (version 1.42; National Institutes of Health, Bethesda, MD; https://imagej.nih.gov/ij/).

### Immunoprecipitation

Cells were lysed in the modified RIPA buffer. Protein extracts were precleaned with protein A/G beads (Invitrogen, catalog No. 1969807) for 30 min. After the centrifugation of protein extracts at 4500 ×*g* for 2 min, supernatants were incubated with 2 µL of anti-nucleolin antibody overnight at 4 °C. After the incubation, supernatants were incubated with protein A/G beads for 1 h. Pellets were washed four times with RIPA buffer, resuspended in it, and analyzed using 10% SDS-polyacrylamide gel electrophoresis (PAGE).

### Separation of subcellular fractionation

Cells were lysed with a fractionation buffer containing 250 mM sucrose, 20 mM HEPES (pH 7.4), 10 mM potassium chloride, 1.5 mM magnesium chloride, 1 mM ethylenediaminetetraacetic acid, 1 mM dithiothreitol, and protease inhibitor cocktail. Nuclear pellets were obtained by centrifugation at 720 ×*g*. Supernatant were collected and centrifuged again at 10,000 ×*g* to remove organelle components. To obtain the cell membrane fraction, supernatants were centrifuged at 100,000 ×*g* for 1 h. Nuclear pellets and membrane pellets were dissolved in RIPA buffer^50^.

### Proliferation assay

Cell proliferation was measured by BrdU incorporation by using a proliferation assay kit (catalog No. 1164-7229001; Roche, Basel, Switzerland) according to the manufacturer’s instructions. PASMCs were plated in 96-well plates (5 × 10^3^ cells/well). After 24 h, PASMCs were starved for 48 h in the serum-free medium, and afterward, this medium was replaced with Dulbecco’s modified eagle medium (DMEM) containing 1% fetal bovine serum (FBS). Cells were treated with 10 µM AS1411 or PBS 2 h before the stimulation with midkine (100 ng/mL) or vehicle, and they were exposed to normoxic or hypoxic conditions (FiO_2_ 0.03). BrdU label solution was added to each well 16 h before the analysis^51^. Denaturing solution was added to each well and cells were incubated for 30 min at room temperature. Afterward, HRP-conjugated anti-BrdU antibody was added to each well and cells were incubated for 1 h at room temperature. The absorbance was read at 450–655 nm on a Benchmark microplate reader (Bio-Rad) and normalized to vehicle control levels.

### Migration assay

PASMCs migration was evaluated using the scratch assay^48^. PASMCs were seeded into 24-well collagen coated dish (1.5 × 10^5^/well). Twenty-four hours after the incubation, PASMCs were starved in serum-free medium for 48 h, a linear wound was created using p1000 pipette tips, and medium was replaced with DMEM containing 0.1% FBS. AS1411 (10 µM) was added 2 h before midkine stimulation and cells were exposed to either normoxia or hypoxia (FiO_2_ 0.03) for 24 h. The scratched area covered by the cells was measured using ImageJ software and normalized to the level of control.

### Statistical analysis

All values are expressed as mean ± standard error (SE), except in Table 1, where the data are presented as mean ± standard deviation (SD). P values for pairwise comparisons of groups were calculated from the Student t distribution. Multiple comparisons between groups were analyzed by ANOVA followed by Tukey’s Honest Significant Difference test. P-values <0.05 were considered statistically significant. Statistical analyses were performed using R version 3.0.1.

## Supplementary information

Supplementary Information.

Supplementary Information 2.
