# Supplementary material for: Growth Factor Midkine Aggravates Pulmonary Arterial Hypertension via Surface Nucleolin
Source: Sci Rep. 2020 Jun 25;10:10345. doi: 10.1038/s41598-020-67217-w (PMC7316741; doi:10.1038/s41598-020-67217-w)
Supplement: Supplementary file 1 — Supplementary Information. [file 41598_2020_67217_MOESM1_ESM.docx]

**Growth Factor Midkine Aggravates Pulmonary Arterial Hypertension via Surface Nucleolin**

Daisuke Kinoshita^1^, Tetsuro Shishido^1*^, Tetsuya Takahashi^1^, Miyuki Yokoyama^1^, Takayuki Sugai^1^, Ken Watanabe^1^, Harutoshi Tamura^1^, Satoshi Nishiyama^1^, Hiroki Takahashi^1^, Takanori Arimoto^1^, Takuya Miyamoto^1^, Tetsu Watanabe^1^, Satoshi Kishida^2^, Kenji Kadomatsu^2^, Jun-ichi Abe^3^, Yasuchika Takeishi^4^, Tsuneo Konta^1^, Isao Kubota^1^ and Masafumi Watanabe^1^

^1^The Department of Cardiology, Pulmonology, and Nephrology, Yamagata University School of Medicine, Yamagata, Japan

^2^Department of Biochemistry, Nagoya University Graduate School of Medicine, Aichi, Japan

^3^Department of Cardiology - Research, Division of Internal Medicine, The University of Texas MD Anderson Cancer Center, Houston, TX

^4^Department of Cardiology and Hematology, Fukushima Medical University, Fukushima, Japan

***Address for correspondence:** Tetsuro Shishido

Department of Cardiology, Pulmonology, and Nephrology,

Yamagata University School of Medicine,

2-2-2 Iida-nishi, Yamagata, 990-9585, Japan

Phone: +81-23-628-5302; Fax: +81-23-628-5305; E-mail: tshishid@med.id.yamagata-u.ac.jp

**Figure Legends:**

Figure S1. Full length of blots in Figure 1c

Uncropped original image. Membranes are often cut to enable blotting for multiple antibodies. Immunoblots are surrounded by a black line to indicate borders of the blots. Specific regions of the original blots cropped for the figures are denoted using dotted lines.

Figure S2. Full length of blots in Figure 2f

Uncropped original image. Membranes are often cut to enable blotting for multiple antibodies. Immunoblots are surrounded by a black line to indicate borders of the blots. Specific regions of the original blots cropped for the figures are denoted using dotted lines.

Figure S3. Full length of blots in Figure 2g

Uncropped original image. Membranes are often cut to enable blotting for multiple antibodies. Immunoblots are surrounded by a black line to indicate borders of the blots. Specific regions of the original blots cropped for the figures are denoted using dotted lines.

Figure S4. Full length of blots in Figure 3d

Uncropped original image. Membranes are often cut to enable blotting for multiple antibodies. Immunoblots are surrounded by a black line to indicate borders of the blots. Specific regions of the original blots cropped for the figures are denoted using dotted lines.

Figure S5. Full length of blots in Figure 3e

Uncropped original image. Membranes are often cut to enable blotting for multiple antibodies. Immunoblots are surrounded by a black line to indicate borders of the blots. Specific regions of the original blots cropped for the figures are denoted using dotted lines.

Figure S6. Full length of blots in Figure 3f

Uncropped original image. Membranes are often cut to enable blotting for multiple antibodies. Immunoblots are surrounded by a black line to indicate borders of the blots. Specific regions of the original blots cropped for the figures are denoted using dotted lines.

Figure S7. Full length of blots in Figure 4a

Uncropped original image. Membranes are often cut to enable blotting for multiple antibodies. Immunoblots are surrounded by a black line to indicate borders of the blots. Specific regions of the original blots cropped for the figures are denoted using dotted lines.

Figure S8. Full length of blots in Figure 4b

Uncropped original image. Membranes are often cut to enable blotting for multiple antibodies. Immunoblots are surrounded by a black line to indicate borders of the blots. Specific regions of the original blots cropped for the figures are denoted using dotted lines.

Figure S9. Full length of blots in Figure 4e

Uncropped original image. Membranes are often cut to enable blotting for multiple antibodies. Immunoblots are surrounded by a black line to indicate borders of the blots. Specific regions of the original blots cropped for the figures are denoted using dotted lines.

Figure S10. Full length of blots in Figure 4f

Uncropped original image. Membranes are often cut to enable blotting for multiple antibodies. Immunoblots are surrounded by a black line to indicate borders of the blots. Specific regions of the original blots cropped for the figures are denoted using dotted lines.

Figure S11. Full length of blots in Figure 5a

Uncropped original image. Membranes are often cut to enable blotting for multiple antibodies. Immunoblots are surrounded by a black line to indicate borders of the blots. Specific regions of the original blots cropped for the figures are denoted using dotted lines.

Figure S12. Full length of blots in Figure 5b

Uncropped original image. Membranes are often cut to enable blotting for multiple antibodies. Immunoblots are surrounded by a black line to indicate borders of the blots. Specific regions of the original blots cropped for the figures are denoted using dotted lines.

Figure S13. Full length of blots in Figure 5f

Uncropped original image. Membranes are often cut to enable blotting for multiple antibodies. Immunoblots are surrounded by a black line to indicate borders of the blots. Specific regions of the original blots cropped for the figures are denoted using dotted lines.
