# Supplementary material for: Growth Factor Midkine Aggravates Pulmonary Arterial Hypertension via Surface Nucleolin
Source: Sci Rep. 2020 Jun 25;10:10345. doi: 10.1038/s41598-020-67217-w (PMC7316741; doi:10.1038/s41598-020-67217-w)

Figure S1 (Figure 1c)

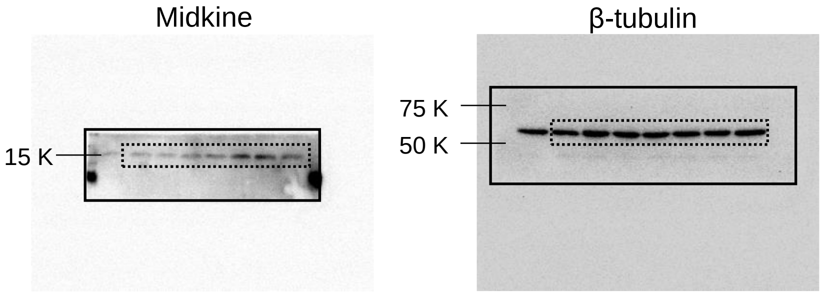

Figure S2 (Figure 2f)

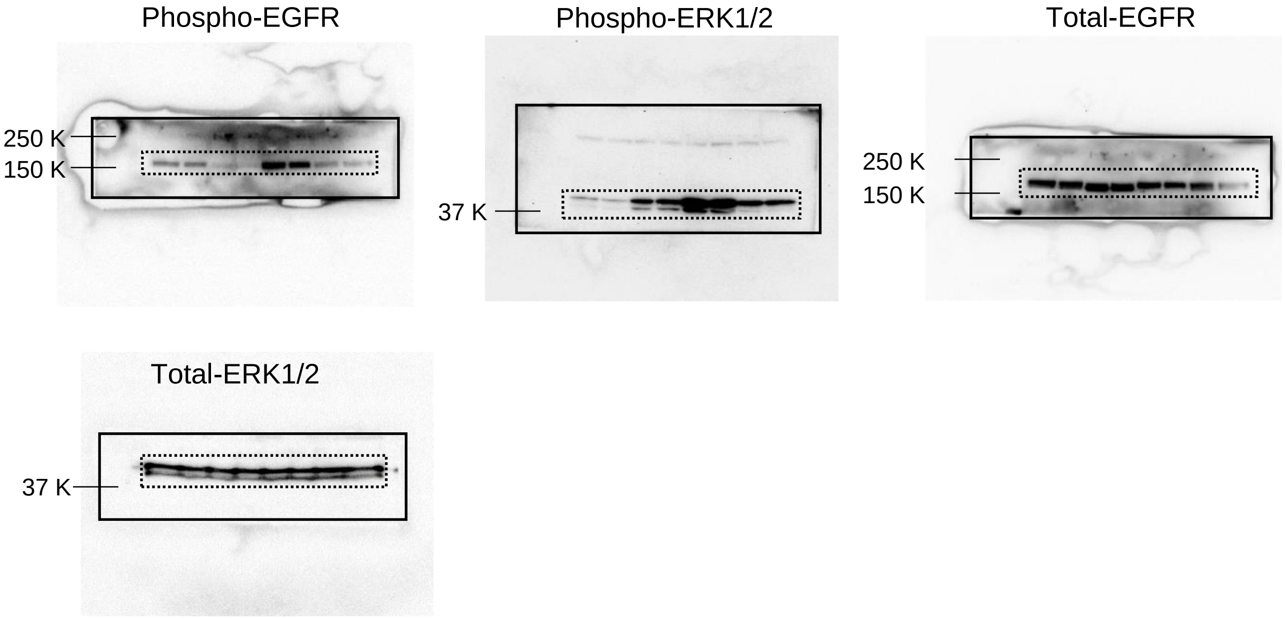

Figure S3 (Figure 2g)

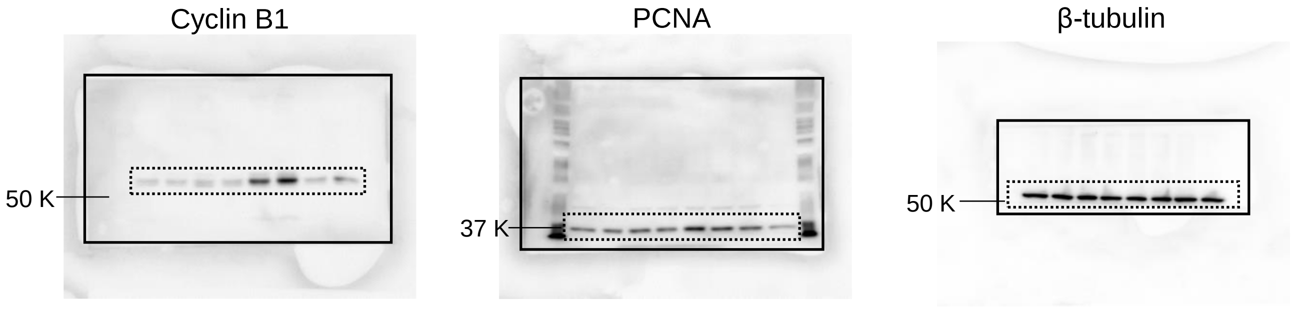

Figure S4 (Figure 3d)

Phospho-EGFR

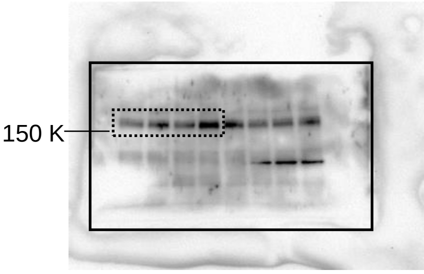

Phospho-ERK1/2

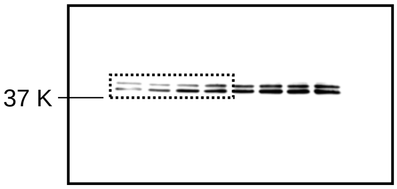

PCNA

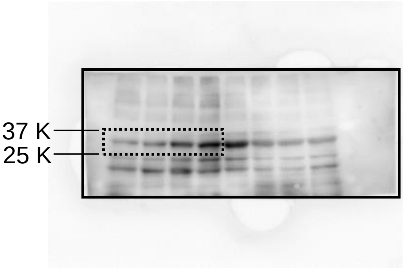

Total-EGFR

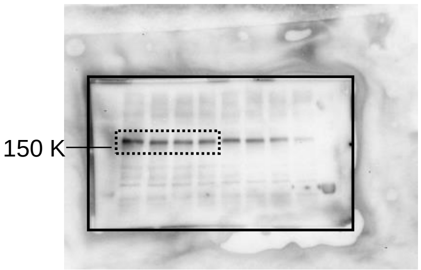

Total-ERK1/2

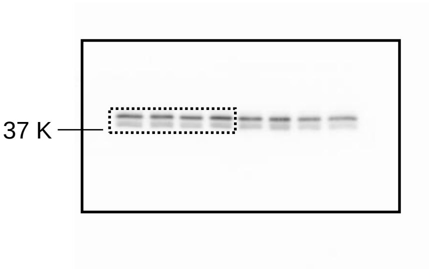

β-tubulin

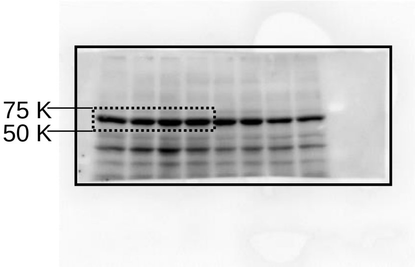

37 K —

Figure S5 (Figure 3e)

Phospho-EGFR

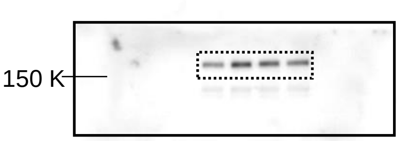

Phospho-ERK1/2

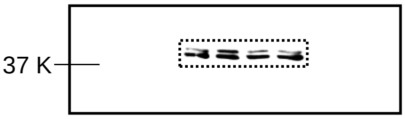

Phospho-ERK1/2 (short exposure)

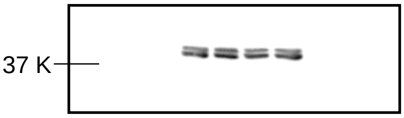

PCNA

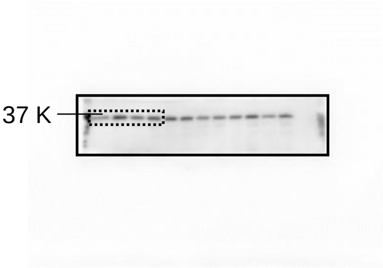

Total-EGFR

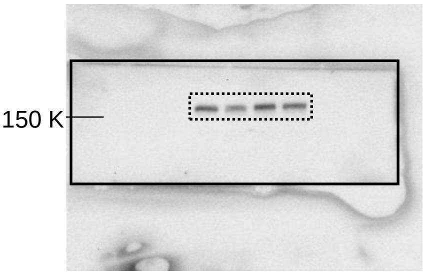

Total-ERK1/2

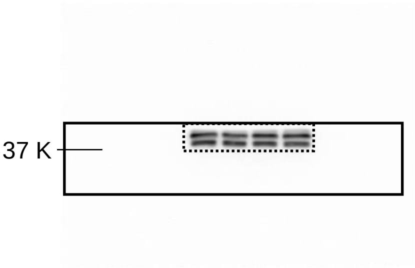

β-tubulin

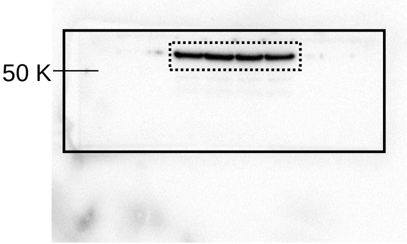

NCL

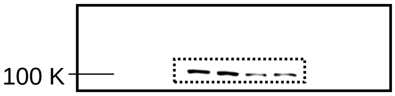

Figure S6 (Figure 3f)

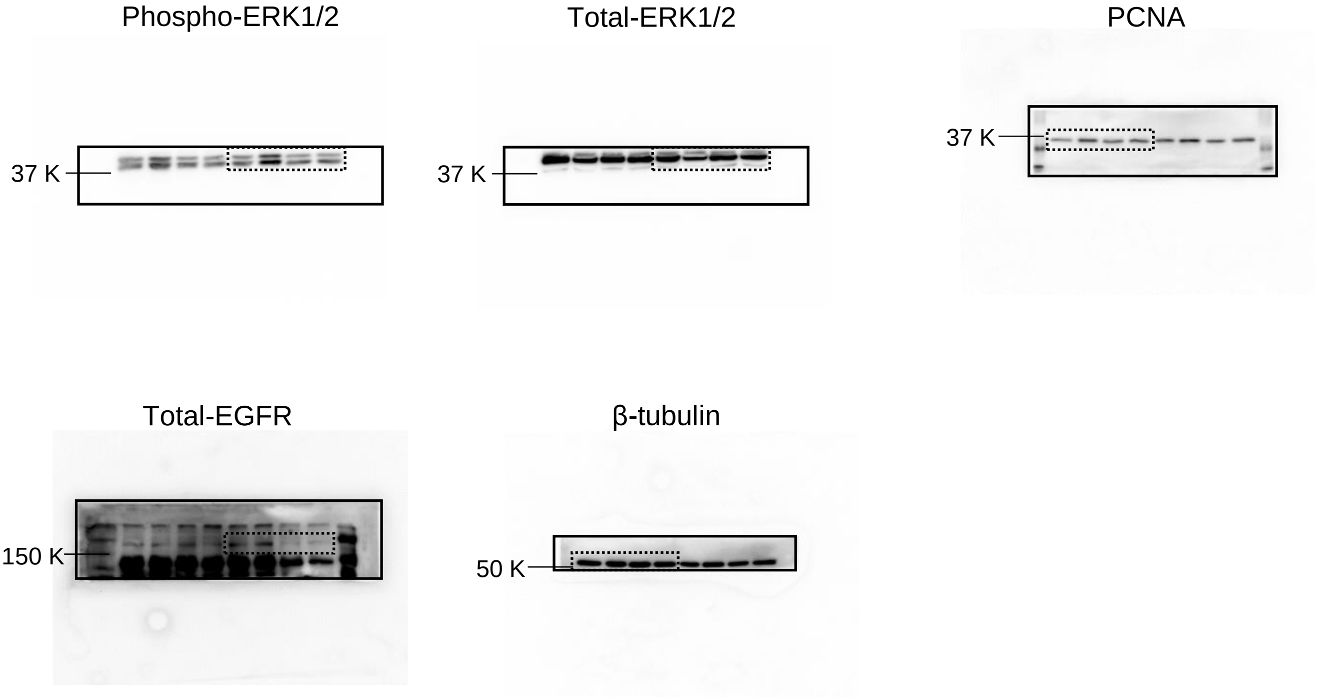

Figure S7 (Figure 4a)

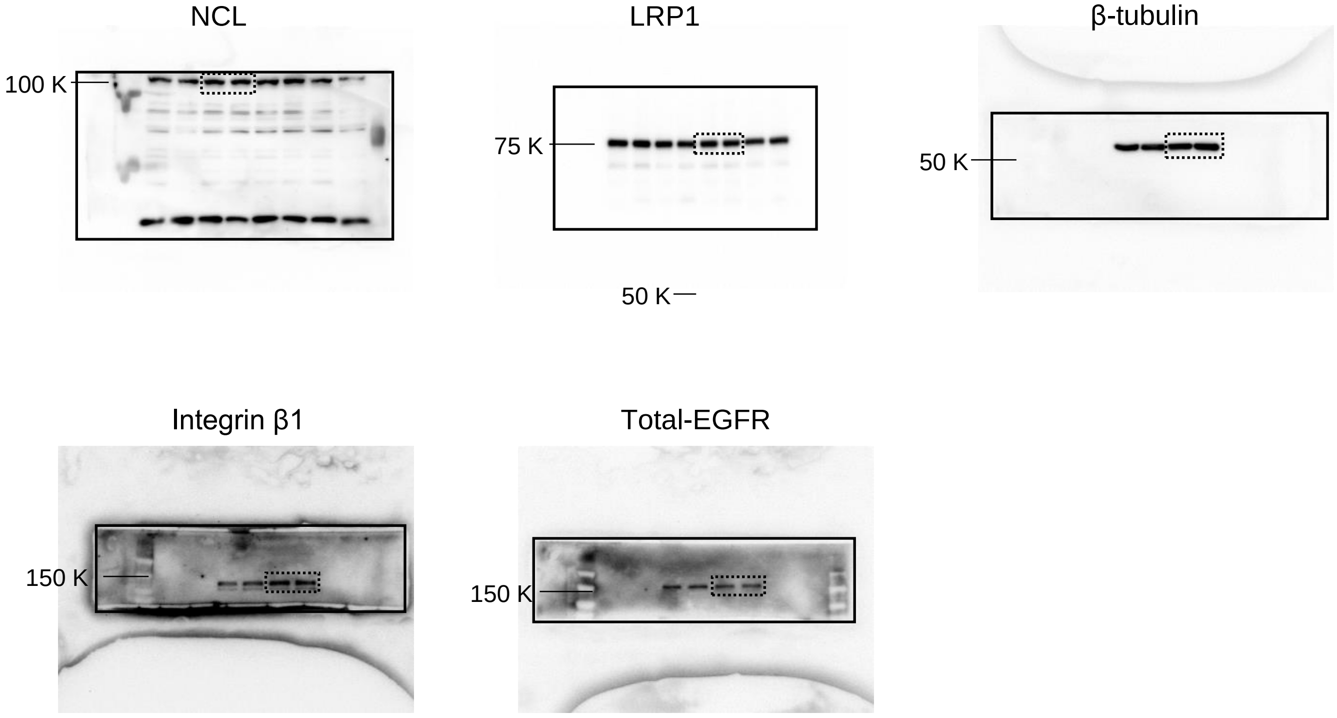

Figure S8 (Figure 4b)

NCL

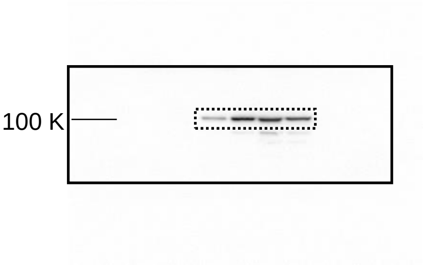

LRP1

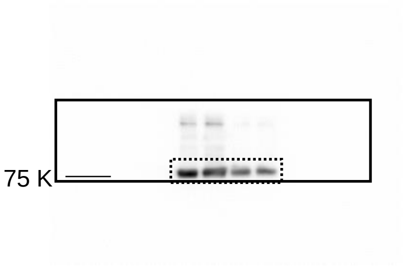

Lamin B1

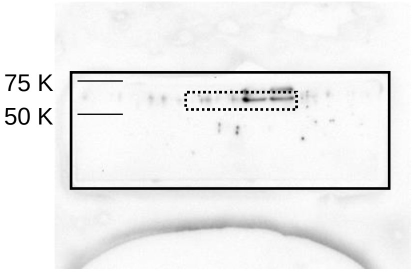

Integrin β1

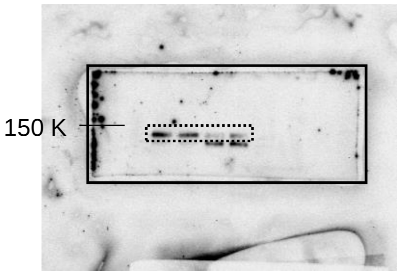

Na/K ATPase

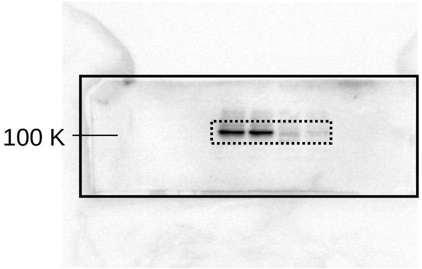

Figure S9 (Figure 4e)

Midkine

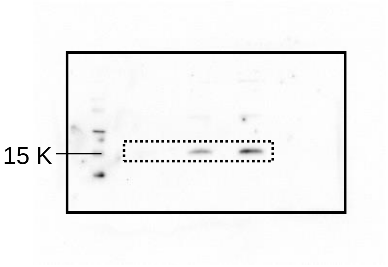

NCL

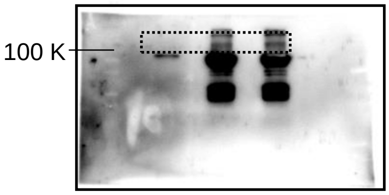

Figure S10 (Figure 4f)

Total-EGFR

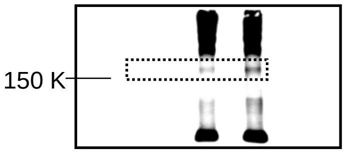

Total-EGFR (short exposure)

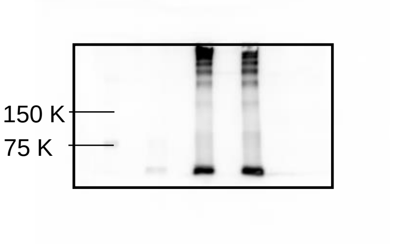

NCL

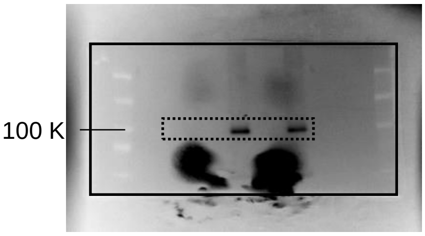

Figure S11 (Figure 5a)

Midkine

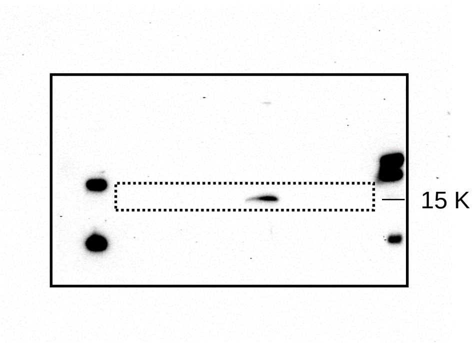

NCL

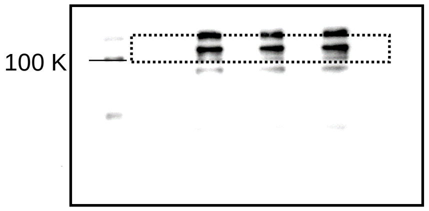

Figure S12 (Figure 5b)

Phospho-EGFR

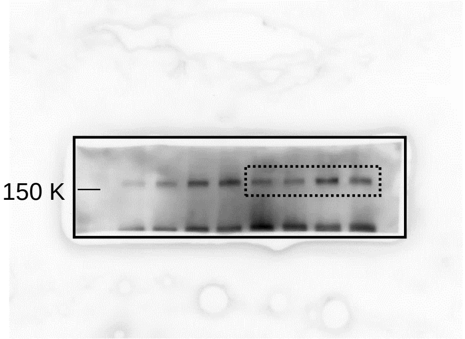

Phospho-ERK1/2

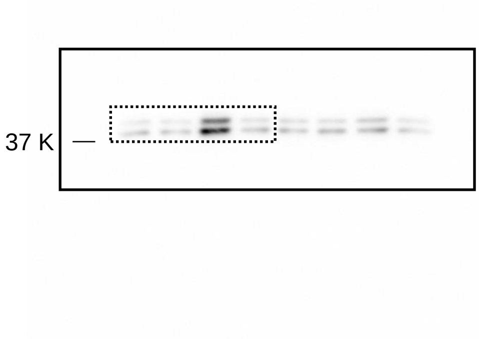

Total-EGFR

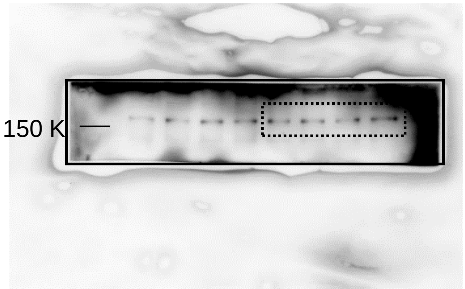

Total-ERK1/2

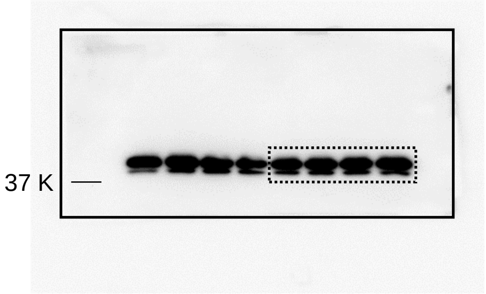

Figure S13 (Figure 5f)

Midkine

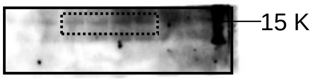

Midkine (short exposure)

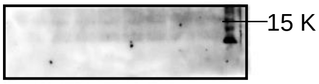

β-tubulin

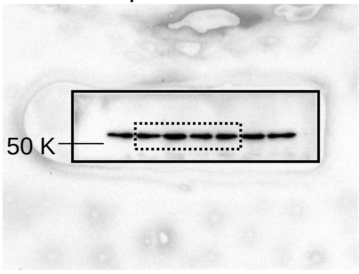

Supplement: Supplementary file 2 — Supplementary Information 2. [file 41598_2020_67217_MOESM2_ESM.pdf]
